# Hepatitis C virus infection and risk factors among patients and health-care workers of Ain Shams University hospitals, Cairo, Egypt

**DOI:** 10.1371/journal.pone.0246836

**Published:** 2021-02-08

**Authors:** Wagida A. Anwar, Maha El Gaafary, Samia A. Girgis, Mona Rafik, Wafaa M. Hussein, Dalia Sos, Isis M. Mossad, Arnaud Fontanet, Laura Temime

**Affiliations:** 1 Faculty of Medicine, Department of Community, Environmental and Occupational Medicine, Ain Shams University, Cairo, Egypt; 2 Faculty of Medicine, Department of Clinical Pathology, Ain Shams University, Cairo, Egypt; 3 Emerging diseases epidemiology Unit, Institut Pasteur, Paris, France; 4 PACRI Unit, Institut Pasteur, Conservatoire national des arts et métiers, Paris, France; 5 Modélisation, Epidémiologie et Surveillance des Risques Sanitaires (MESuRS), Conservatoire national des arts et métiers, Paris, France; Macfarlane Burnet Institute for Medical Research and Public Health, Australia, AUSTRALIA

## Abstract

**Background:**

Hospitals are suspected of playing a key role in HCV epidemic dynamics in Egypt. This work aimed at assessing HCV prevalence and associated risk factors in patients and health-care workers (HCWs) of Ain Shams University (ASU) hospitals in Cairo.

**Methods:**

We included 500 patients admitted to the internal medicine or surgery hospital from February to July, 2017, as well as 50 HCWs working in these same hospitals. Participants were screened for anti-HCV antibodies and HCV RNA. A questionnaire was administered to collect data on demographic characteristics and medical/surgical history. For HCWs, questions on occupational exposures and infection control practices were also included.

**Results:**

The overall prevalence of anti-HCV antibodies was 19.80% (95% CI: 16.54–23.52) among participating patients, and 8.00% (95% CI: 0.48–15.52) among participating HCWs. In HCWs, the only risk factors significantly associated with anti-HCV antibodies were age and profession, with higher prevalence in older HCWs and those working as cleaners or porters. In patients, in a multivariate logistic regression, age over 50 (aOR: 3.4 [1.9–5.8]), living outside Cairo (aOR: 2.1 [1.2–3.4]), admission for liver or gastro-intestinal complaints (aOR: 4.2 [1.8–9.9]), and history of receiving parenteral anti-schistosomiasis treatment (aOR: 2.7 [1.2–5.9]) were found associated with anti-HCV antibodies.

**Conclusions:**

While HCV prevalence among patients has decreased since the last survey performed within ASU hospitals in 2008, it is still significantly higher than in the general population. These results may help better control further HCV spread within healthcare settings in Egypt by identifying at-risk patient profiles upon admission.

## Introduction

Hepatitis C virus (HCV) is a blood-borne virus and its major routes of transmission include blood transfusion, medical injections and procedures, and injection drug use [1].

Worldwide, according to the World Health Organization, 71 million people are estimated to be chronically infected with HCV and over 350,000 die annually from liver disease caused by HCV [2,3].

Egypt is the country with the highest HCV prevalence worldwide–antibody prevalence in Egypt was estimated at 11.9% in a 2018 meta-analysis [4], and the Egyptian Demographic and Health Surveys (EDHS) measured antibody prevalence among the adult population aged 15–59 years at 14.7% in 2009 [5] and at 10.0% in 2015 [6], substantially higher than the global levels estimated at 1.4% by WHO [2]. This results in 6 million chronically infected individuals in Egypt [7].

The origin of the Egyptian HCV epidemic has been attributed to campaigns for mass treatment of schistosomiasis which took place between 1960 and 1984, when intravenous injections with antimony salts were given to 3–5 million people older than 6 years of age. Insufficient sterilization of needles and syringes was considered to be responsible for HCV transmission at that time [8]. Since HCV-infected individuals may survive for more than 30 years, many persons infected during that period are still alive today. They form an aging cohort from whom HCV is still spreading to younger generations through a variety of healthcare procedures such as injections, intravenous catheterization, surgery, etc. [9,10].

Hospitals are suspected of playing a key role in the continuing HCV transmission in Egypt, with previous hospitalization known as a major risk factor for HCV infection in this country [11]. An elevated risk of HCV acquisition among Egyptian hospitalized patients has indeed been documented, for instance in dialysis patients [12]. This is due to the combination of the high frequency of invasive procedures performed on patients, with documented evidence of imperfect compliance with standard precaution recommendations [13], and the over-representation of HCV-infected individuals among hospital patients. For instance, a study conducted in 2008–10 at Ain Shams University hospitals in Cairo found a 42% prevalence of HCV antibodies among the hospital’s patients, also resulting in a high risk of HCV exposure for the hospital staff [14].

In this context, the objectives of this work were to determine an updated estimate of the prevalence of HCV infection among healthcare workers and adult hospitalized patients in Ain Shams University hospitals, as well as the associated risk factors.

## Methods

An observational cross-sectional study was conducted at Ain Shams University Hospitals, as part of the larger project entitled Investigative Mathematical Modeling of Hospital Transmission of Hepatitis C (ANRS 12320 IMMHoTHep). The study targeted health care workers (HCWs) and hospitalized patients at Ain Shams University Hospitals. Study participants were recruited from the surgical and internal medicine hospitals over a 22-week period from February to July, 2017.

### Patients

The inclusion criteria for the participating patients included: age ≥ 21 years, hospital stay exceeding 24 hours, and being admitted to either the surgical or the internal medicine hospital. A sample of 500 hospitalized patients (274 from the surgical hospital and 226 from the internal medicine hospital, in proportion with annual admissions to these respective hospitals) were recruited over the 22-week period from either the outpatient clinic and or the emergency room. Total numbers of recruited patients from each place were distributed to reflect the relative importance of these modes of admission into the surgical and internal medicine hospitals. Each week, two days (from Sunday to Saturday) and times (between 8 AM and 5 PM) were randomly selected to recruit patients. At the first selected time and day, the list of patients admitted within the last 24 hours to the surgery hospital via the surgery outpatient clinic was retrieved, as well as the list of patients admitted to the internal medicine via the internal medicine outpatient clinic. Study participation was then proposed consecutively to the patients on these two lists, starting from the most recently admitted patient and going retrospectively in time until the recruitment target for the week was met for each hospital (4 to 6 patients/hospital/week). Similarly, at the second time and day, the list of patients admitted via the emergency room within the last 24 hours was retrieved. Study participation was then proposed consecutively to patients admitted to the surgery hospital on the one hand, and to patients admitted to the internal medicine hospital on the other hand, going backwards in time until the recruitment targets for the week were met for each hospital (6 to 7 patients/hospital/week).

### HCWs

The inclusion criteria for the participating HCWs included: age ≥ 21 years and working in either the surgical or the internal medicine hospital. A sample of 50 HCWs were recruited over the same 22-week period (27 from the surgical hospital and 23 from the internal medicine hospital, in proportion with annual patient admissions to these respective hospitals). Within each hospital, a pre-defined number of staff from each profession to recruit, along with their planned weeks of recruitment, was determined so that total recruited HCWs reproduced observed percentages of HCWs from each profession, based on administrative 2017 data. Randomly ordered lists of staff from each profession were generated from human resources databases. On each odd week, 2 to 3 HCWs were recruited by proposing study participation consecutively to HCWs of the professions selected for that week in each hospital, until the recruitment targets for the week were met.

### Data collection tools

A structured questionnaire was administered upon inclusion into the study including demographic characteristics of the respondent (whether patient or HCW), medical/surgical history stressing on invasive medical procedures, history of treatment with parenteral anti-schistosomiasis, and previous diagnosis of HCV infection. For HCWs, questions on occupational exposures and infection control practices were also included. These questionnaires were first tested in a pilot study that took place in January 2017 in the same hospitals as the main study.

A blood sample of 5 mL in two tubes was withdrawn from both patients and HCWs to assess the presence of anti-HCV antibodies (chemiluminescent assay) in all study participants at the time of study admission. Participants who tested positive for anti-HCV antibodies were further tested for HCV RNA (RT-PCR).

### Ethical considerations

Approval from the Institutional Review Board of the Faculty of Medicine of Ain Shams University (Ain Shams-IRB) was obtained. Questionnaires were anonymous and confidentiality of data was ensured, with only persons involved in the study management having access to the medical files of included participants. A written consent was obtained from all persons participating in the research after explanation of the objectives, nature, benefits and constraints of the research. Study participants had the right to withdraw from the research at any time. Ethical approval from Sheffield University, School of Health and Related Research (ScHARR) was also obtained. All guidelines, including treating data as confidential and not making effort to identify participants, were respected. Good clinical practice certification was provided to all investigators after the conduction of a training session.

### Sample size

As mentioned before, this work is actually a sub-study of the IMMHoTHep study that aims at better understanding patient care pathways within the hospital, with a focus on invasive procedures and moves between hospital wards and departments. Sample size was chosen in the context of this larger study, for which discussions with health practitioners from Ain Shams hospitals suggested that including 500 patients over a 6-month period would allow us to observe a wide array of different patient trajectories. Regarding HCWs, IMMHoTHep mostly aimed at deriving a qualitative picture of their working patterns and infection control practices, based on direct observations sessions; in this regard, only 50 HCWs were included.

### Statistical analysis

Data analysis was performed using the R software (Version 4.0.0). Data analysis included descriptive statistics of the study sample reported as frequency and percentages for categorical variables; and as means and standard deviations for continuous variables. HCV antibody and HCV RNA prevalence with their 95% confidence intervals were estimated. Pearson Chi-square/Fisher exact tests were used to compare HCV status between groups.

Explanatory variables included: age as categorical variable, gender, marital status, place of living, education level, history of parenteral anti-schistosomiasis treatment (PAT), history of surgical intervention; history of invasive medical procedures (intravenous catheterization, sutures, blood transfusion, blood sampling, injection, endoscopy, gastric lavage, cardiac catheterization, dialysis and wound dressing) in participants with history of hospitalization; and history of occupational exposure (sharp cuts, syringe injuries, blood exposure incidents) and infection control practices (gloves, gowns, face masks, goggles) in HCWs.

A logistic regression analysis was carried out to assess the risk factors significantly associated with HCV antibody status in patients. Variables with p-values ≤ 0.05 were introduced simultaneously in a multivariate logistic regression model. The final model was obtained by removing variables with the highest p-values one by one and using the Akaike information criterion (AIC) to assess which model fit the data best. The model with the lowest AIC value was selected as the best model. The goodness of fit of this model was then assessed using the Hosmer-Lemeshow test, and its discriminating power was assessed from the area under the receiver-operating curve (AUC).

Considering the low number of included HCWs, for the HCW data no multivariate analysis was attempted and Firth’s bias reduction method was applied when estimating unadjusted odds-ratios and their confidence intervals from penalized logistic regression [15].

## Results

### Characteristics of participants

Tables 1 and 2 show the characteristics of the 500 patient participants and 50 HCW participants to the study. Overall, 55% of the participants were recruited from the surgery hospital while the remaining 45% were recruited from the internal medicine hospital, as aimed for to reflect relative admission rates to these two hospitals. The patients’ age ranged from 21 to 85 (mean± standard deviation = 45.37 ±16.21), while HCWs were aged 22 to 57 (mean± standard deviation = 36.36 ±10.80). Females comprised 42.40% of the patient participants and 66.00% of HCW participants, and 74.80% of the patient participants (resp. 60.00% of the HCW participants) were married at the time of study.

**Table 1:** Socio-demographic factors associated with HCV antibody status among participating patients.

	Total N = 500	Positive N = 99	Negative N = 401	Crude OR (95%CI)	p-value ^a^
		N (Row %)	N (Row %)		
**Hospital**					<0.001
**Surgery hospital**	274	34 (12.41)	240 (87.59)	Reference	
**Internal medicine hospital**	226	65 (28.76)	161 (71.24)	2.85 (1.80–4.52)*	
**Admission type**					0.172
**Outpatient clinic**	227	51 (22.47)	176 (77.53)	Reference	
**Emergency department**	273	48 (17.58)	225 (82.42)	0.74 (0.47–1.14)	
**Age**					<0.001
**< 50**	285	26 (9.12)	259 (90.88)	Reference	
**≥ 50**	215	73 (33.95)	142 (66.05)	5.12 (3.13–8.38)*	
**Gender**					0.023
**Female**	212	32 (15.09)	180 (84.91)	Reference	
**Male**	288	67 (23.26)	221 (76.74)	1.71 (1.07–2.71)*	
**Marital status**					0.281
**Single**	71	10 (14.08)	61 (85.92)	Reference	
**Married**	374	75 (20.05)	299 (79.95)	1.53 (0.75–3.13)	
**Widow**	41	9 (21.95)	32 (78.05)	1.72 (0.63–4.65)	
**Divorced**	14	5 (35.71)	9 (64.29)	3.39 (0.94–12.21)	
**Place of living**					<0.001
**Cairo**	333	48 (14.41)	285 (85.59)	Reference	
**Other**	167	51 (30.54)	116 (69.46)	2.61 (1.67–4.09)*	
**Education level**					0.001
**Secondary/ University**	200	28 (14)	172 (86)	Reference	
**Preparatory at most**	156	29 (18.59)	127 (81.41)	1.4 (0.8–2.47)	
**Illiterate**	144	42 (29.17)	102 (70.83)	2.53 (1.48–4.33)*	
**Working status**					0.868
**Unemployed/ Retired**	269	54 (20.07)	215 (79.93)	Reference	
**Employed**	231	45 (19.48)	186 (80.52)	0.96 (0.62–1.5)	
**Indication for current admission**					<0.001
**General surgery ^b^**	181	18 (9.94)	163 (90.06)	Reference	
**Special surgery ^b^**	101	15 (14.85)	86 (85.15)	1.58 (0.76–3.29)	
**General IM ^b^**	44	7 (15.91)	37 (84.09)	1.71 (0.67–4.4)	
**Special IM ^b^**	121	27 (22.31)	94 (77.69)	2.6 (1.36–4.97)*	
**Liver/ GIT complaint**	53	32 (60.38)	21 (39.62)	13.8 (6.62–28.77)*	

IM: Internal medicine, **GIT**: Gastro-intestinal.

^a^ Chi2/ Fisher exact test.

^b^ Other than Liver/ GIT symptom/ sign.

* Indicates statistical significance (p<0.05).

**Table 2:** Medical exposures associated with HCV antibody status among participating patients.

	Total N = 500	Positive N = 99	Negative N = 401	Crude OR (95%CI)	P value ^a^
		N (Row %)	N (Row %)		
**PAT**					<0.001
**No**	460	76 (16.52)	384 (83.48)	Reference	
**Yes**	40	23 (57.5)	17 (42.5)	6.84 (3.49–13.4)*	
**Surgery**					0.905
**No**	139	28 (20.14)	111 (79.86)	Reference	
**Yes**	361	71 (19.67)	290 (80.33)	0.97 (0.6–1.58)	
**Intravenous catheter**					0.450
**No**	152	27 (17.76)	125 (82.24)	Reference	
**Yes**	348	72 (20.69)	276 (79.31)	1.21 (0.74–1.97)	
**Sutures**					0.74
**No**	260	50 (19.23)	210 (80.77)	Reference	
**Yes**	240	49 (20.42)	191 (79.58)	1.08 (0.69–1.67)	
**Blood transfusion**					<0.001
**No**	403	66 (16.38)	337 (83.62)	Reference	
**Yes**	97	33 (34.02)	64 (65.98)	2.63 (1.6–4.32)*	
**Blood sample**					0.145
**No**	146	23 (15.75)	123 (84.25)	Reference	
**Yes**	354	76 (21.47)	278 (78.53)	1.46 (0.88–2.44)	
**Injection**					0.432
**No**	194	35 (18.04)	159 (81.96)	Reference	
**Yes**	306	64 (20.92)	242 (79.08)	1.2 (0.76–1.9)	
**Endoscopy**					<0.001
**No**	425	67 (15.76)	358 (84.24)	Reference	
**Yes**	75	32 (42.67)	43 (57.33)	3.98 (2.35–6.73)*	
**Gastric lavage**					<0.001
**No**	486	91 (18.72)	395 (81.28)	Reference	
**Yes**	14	8 (57.14)	6 (42.86)	5.79 (1.96–17.09)*	
**IUD insertion ^b^**					0.376
**No**	134	18 (13.43)	116 (86.57)	Reference	
**Yes**	78	14 (17.95)	64 (82.05)	1.41 (0.66–3.02)	
**Cardiac catheter**					0.253
**No**	480	93 (19.38)	387 (80.63)	Reference	
**Yes**	20	6 (30)	14 (70)	1.78 (0.67–4.77)	
**Dialysis**					1.000
**No**	489	97 (19.84)	392 (80.16)	Reference	
**Yes**	11	2 (18.18)	9 (81.82)	0.9 (0.19–4.22)	
**Wound dressing**					0.864
**No**	279	56 (20.07)	223 (79.93)	Reference	
**Yes**	221	43 (19.46)	178 (80.54)	0.96 (0.62–1.5)	
**Hospitalization**					0.149
**No**	124	19 (15.32)	105 (84.68)	Reference	
**Yes**	376	80 (21.28)	296 (78.72)	1.49 (0.86–2.58)	
**ASU hospital admission**					0.011
**No**	207	34 (16.43)	173 (83.57)	Reference	
**Yes**	169	46 (27.22)	123 (72.78)	1.9 (1.15–3.14)*	
**Public hospital admission**					0.123
**No**	155	39 (25.16)	116 (74.84)	Reference	
**Yes**	221	41 (18.55)	180 (81.45)	0.68 (0.41–1.11)	
**Private hospital admission**					0.293
**No**	294	66 (22.45)	228 (77.55)	Reference	
**Yes**	82	14 (17.07)	68 (82.93)	0.71 (0.38–1.35)	
**Number of hospitalizations ^c^**					0.196
**None**	124	19 (15.32)	105 (84.68)	reference	
**Once**	137	33 (24.09)	104 (75.91)	1.75 (0.94–3.28)	
**> once**	232	44 (18.97)	188 (81.03)	1.29 (0.72–2.33)	

**PAT**: Parenteral anti-schistosomiasis treatment.

^a^ Chi2/ Fisher exact test.

^b^ In females only.

^c^ Seven patients couldn’t remember the number of their past hospitalizations.

* Indicates statistical significance (p<0.05).

**Fig 1:**
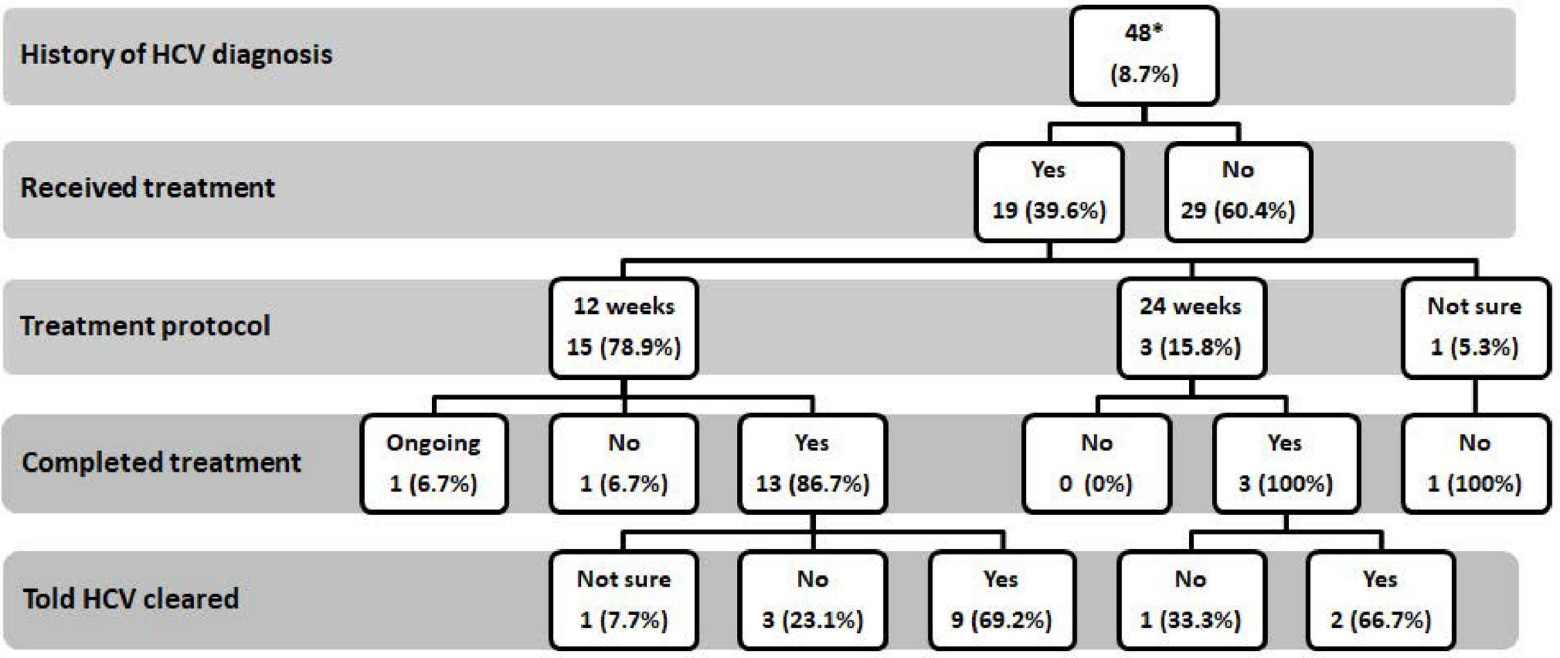
Treatment course of patients who received a previous diagnosis of HCV. * Of the 48 patients with previous HCV diagnosis, 43 (89.58%) tested positive for anti HCV in the current study.

75.20% of patient (resp. 70.00% of HCW) participants had been previously hospitalized and 72.20% (resp. 62.00% of HCW) had a history of surgical intervention. Forty (8.00%) patient participants reported receiving PAT and 48 (9.60%) reported having a previous diagnosis of HCV. Fig 1 shows a flow chart of treatment course of the 48 patients who had received previous HCV diagnosis. None of the HCW participants recalled having received PAT, and none reported having been diagnosed with HCV before the study.

### Prevalence of anti-HCV positive status and association with risk factors

The overall prevalence of anti-HCV antibodies was 19.80% (95% CI: 16.54–23.52) among participating patients, and 8.00% (95% CI: 0.48–15.52) among participating HCWs. Among the 99 patients who were anti-HCV seropositive, 56 (56.57%) had positive viremia, while 2 (50.00%) of the 4 anti-HCV seropositive HCWs had positive viremia, translating as 11.20% of viremic patients (95% CI: 8.44–13.73) and 4.00% of viremic HCWs (95% CI: 0–9.43) overall. Interestingly, a previous HCV infection diagnosis was reported by only 43 of the 99 patients who were found to have anti-HCV antibodies upon hospital admission, and by none of the 4 anti-HCV positive HCWs.

**Fig 2:**
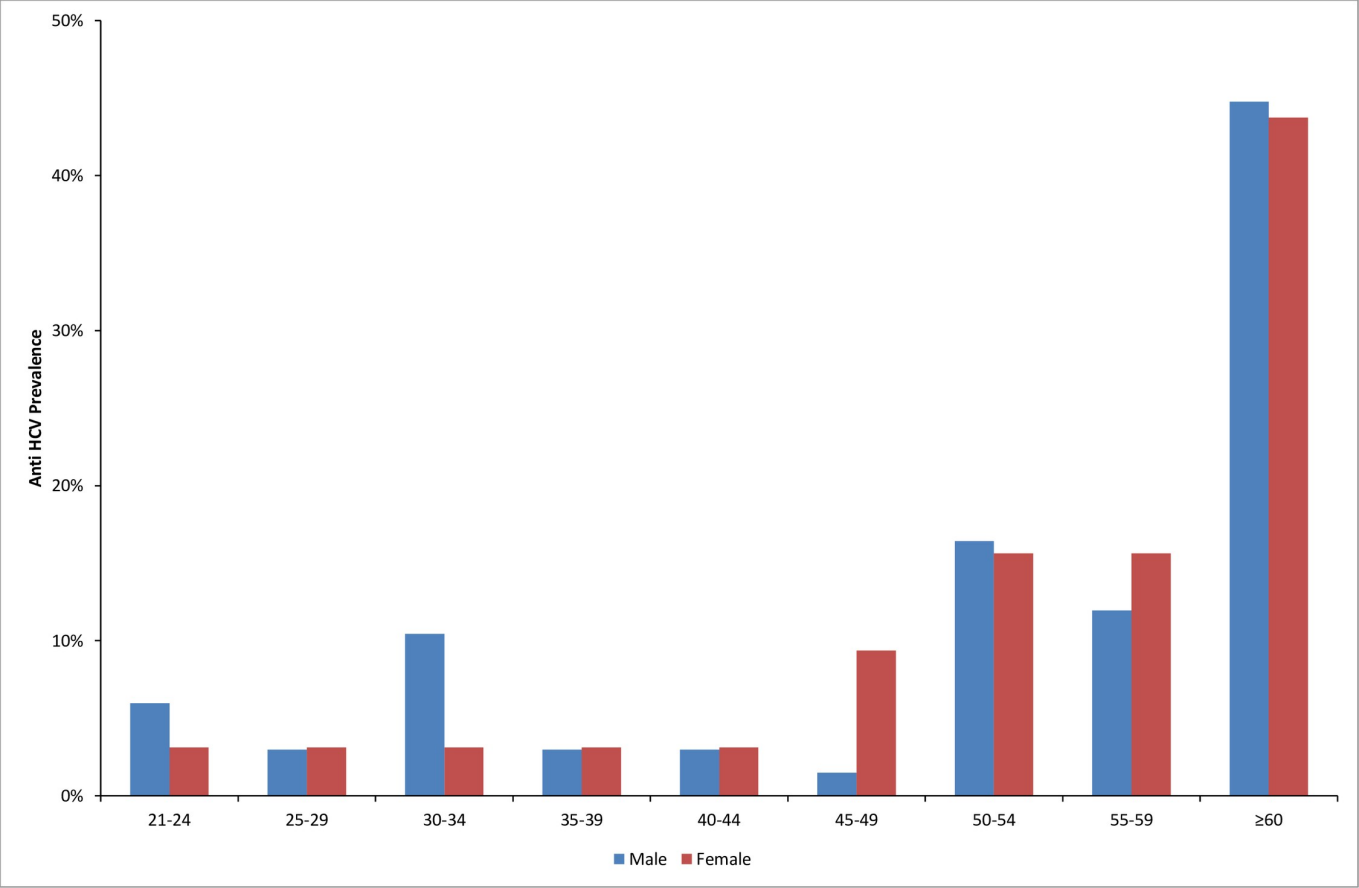
Prevalence of anti-HCV antibodies by sex and age groups among participating patients.

Table 1 shows the association between HCV infection and potential socio-demographic risk factors among patients. Patients admitted to the internal medicine hospital were twice as likely to test positive for anti-HCV antibodies compared to patients admitted to surgery hospital (p < 0.001). The sex-specific prevalence was significantly higher among males (23.26%) than in females 15.09% (p = 0.023). Older age was also significantly associated with positive HCV status (p < 0.001). The mean age of anti-HCV positive patients was 54.35 ±14.46 years, while the mean age of anti-HCV negative patients was 43.16 ±15.86. The most affected age group was the age group ≥ 60 years, especially among men, who had an anti-HCV prevalence of 44.78% (95% CI: 33.48–56.64). Fig 2 depicts the prevalence of anti-HCV positivity by sex and age groups. Education level was significantly associated with HCV infection (p = 0.002), with the highest prevalence of anti-HCV seropositivity observed among illiterates at 42.42% (95% CI: 33.15–52.26).

Table 2 shows the association between HCV infection and the history of medical exposures among patients. Patients with a history of anti-schistosomiasis treatment (PAT) had a nearly 7-fold increase in the odds of being anti-HCV positive (p < 0.001). Histories of having had a gastric lavage, having had an endoscopy and receiving blood transfusion were also significantly associated with anti-HCV positivity. A history of hospitalization was not associated with HCV status; however, a relatively higher proportion (58.33%) of patients with a previous admission to ASU hospitals tested positive for anti-HCV antibodies compared to those who had never been admitted to ASU hospitals (p = 0.011).

Tables 3 and 4 present the association between potential socio-demographic and occupational risk factors and HCV infection among participating HCWs. The only factors significantly associated with anti-HCV seropositivity were older age (p = 0.007) and profession (p = 0.002). The mean age of anti-HCV positive HCWs was 51.25 ± 5.38 years, while the mean age of negative HCWs was 35.07 ± 10.18 years. All anti-HCV positive participating HCWs were cleaners or porters, with 40.00% (4 out of 10) participating HCWs from these professions being positive. None of the anti-HCV positive HCWs reported always wearing gloves before contact with non-intact skin (vs. 43.48% of negative HCWs, p = 0.14), or gowns in case of potential contact with body fluids or blood (vs. 54.35% of negative HCWs, p = 0.11). Finally, a higher proportion of anti-HCV positive HCWs had a history of injuring themselves with sharp objects at work (75.00% vs. 34.78% of negative HCWs, p = 0.15). No past medical/hospitalization/surgery exposure was found to be associated with HCV status in HCWs.

**Table 3:** Socio-demographic factors associated with HCV antibody status among participating HCWs.

	Total N = 50	Positive N = 4	Negative N = 46	Crude OR (95%CI)^a^	p-value^b^
		N (Row %)	N (Row %)		
**Age**					0.007
**< 50**	43	1 (2.33)	42 (97.67)	Reference	
**≥ 50**	7	3 (42.86)	4 (67.14)	22.04 (2.39–203.57)*	
**Gender**					1.000
**Female**	33	3 (5.88)	30 (94.12)	Reference	
**Male**	17	1 (9.09)	16 (90.91)	0.79 (0.10–6.18)	
**Marital status**					0.641
**Single, widowed or divorced**	20	1 (5.00)	19 (95.00)	Reference	
**Married**	30	3 (10.00)	27 (90.00)	1.65 (0.21–12.76)	
**Place of living**					0.560
**Cairo**	41	3 (7.32)	38 (92.68)	Reference	
**Other**	9	1 (11.11)	8 (88.89)	1.94 (0.23–16.48)	

^a^ Computed using Firth’s penalized logistic regression.

^b^ Based on a Fisher exact test.

* Indicates statistical significance (p<0.05).

**Table 4:** Occupational exposure factors associated with HCV antibody status among participating HCWs.

	Total N = 50	Positive N = 4	Negative N = 46	Crude OR (95%CI)^a^	p-value^b^
		N (Row %)	N (Row %)		
**Hospital**					0.614
**Surgery hospital**	27	3 (1.11)	24 (88.89)	Reference	
**Internal medicine hospital**	23	1 (4.35)	22 (95.65)	0.47 (0.06–3.58)	
**Profession**					0.002
**Doctor (resident or house officer)**	13	0 (0.00)	13 (100.00)	Reference	
**Cleaner or porter**	10	4 (40.00)	6 (60.00)	18.69 (0.77–453.54) *	
**Nurse or nurse’s aide**	22	0 (0.00)	22 (100.00)	0.60 (0.01–36.12)	
**Technician**	5	0 (0.00)	5 (100.00)	2.45 (0.03–184.03)	
**Other profession (outside ASU hospital)**					0.353
**No**	45	3 (6.67)	42 (93.33)	Reference	
**Yes**	5	1 (20.00)	4 (80.00)	4.0 (0.40–40.56)	
**Gloves always worn before contact with blood**					1
**No**	30	2 (6.67)	28 (93.33)	Reference	
**Yes**	20	2 (10.00)	18 (90.00)	1.54 (0.23–10.16)	
**Gloves always worn before contact with body fluids**					1
**No**	31	3 (9.68)	28 (90.32)	Reference	
**Yes**	19	1 (5.26)	18 (94.74)	0.66 (0.09–5.11)	
**Gloves always worn before contact with mucous membranes**					1
**No**	31	3 (9.68)	28 (90.32)	Reference	
**Yes**	19	1 (5.26)	18 (94.74)	0.66 (0.09–5.11)	
**Gloves always worn before contact with non-intact skin**					0.14
**No**	30	4 (13.33)	26 (86.67)	Reference	
**Yes**	20	0 (0.00)	20 (100.00)	0.14 (0.01–3.03)	
**Gloves always worn before contact with contaminated equipment**					0.28
**No**	32	4 (12.50)	28 (87.50)	Reference	
**Yes**	18	0 (0.00)	18 (100.00)	0.17 (0.01–3.64)	
**Gown always worn in case of potential contact with blood or body fluids**					0.11
**No**	25	4 (16.00)	21 (84.00)	Reference	
**Yes**	25	0 (0.00)	25 (25.00)	0.09 (0.00–1.95)	
**Goggles always worn in case of potential contact with blood or body fluids**					0.41
**No**	44	3 (6.82)	41 (93.18)	Reference	
**Yes**	6	1 (16.67)	5 (83.33)	3.23 (0.34–30.39)	
**Face mask always worn in case of potential contact with blood or body fluids**					1
**No**	41	4 (9.76)	37 (90.24)	Reference	
**Yes**	9	0 (0.00)	9 (100.00)	0.44 (0.02–10.30)	
**History of syringe injury at work**					1
**No**	16	1 (6.25)	15 (93.75)	Reference	
**Yes**	34	3 (8.82)	31 (91.18)	1.15 (0.15–9.00)	
**History of injury with a sharp object at work**					0.15
**No**	31	1 (3.23)	30 (96.77)	Reference	
**Yes**	19	3 (15.79)	16 (84.21)	4.31 (0.56–33.27)	
**History of blood exposure of non-intact skin at work**					1
**No**	36	3 (8.33)	33 (91.67)	Reference	
**Yes**	14	1 (7.14)	13 (92.86)	1.06 (0.13–8.45)	
**Awareness of HCV**					1
**No**	3	0 (0.00)	3 (100.0)	Reference	
**Yes**	47	4 (8.51)	43 (91.49)	0.72 (0.02–25.45)	

^a^ Computed using Firth’s penalized logistic regression.

^b^ Based on a Fisher exact test.

* Indicates statistical significance (p<0.05).

### Multivariable analysis of risk factors for anti-HCV status of patients

Eleven variables that had level of significance 0.05 or less in the bivariate analysis were included in the multivariate logistic regression model. The initial full model included: hospital type; age categories; gender; place of living; education level; indication for current admission; history of receiving PAT; history of receiving blood transfusion, history of having gastric lavage, history of endoscopy and history of past ASU hospital admission.

During backward elimination, history of having gastric lavage was removed first, followed by history of having endoscopy, then education, then gender, and finally hospital type. The final best model to explain HCV status, presented in Table 5, included: age; place of living; indication for current admission; history of receiving PAT; history of blood transfusion and history of past ASU hospital admission. Significant association was observed between anti-HCV seropositivy and older age, living outside Cairo, Liver/GIT-related current admission and history of PAT. The model had good fit according to the Hosmer‐Lemeshow test (P = 0.51), as well as good discrimination (AUC = 0.79 [0.74–0.84]).

**Table 5:** Multiple logistic regression model describing variables associated with anti-HCV status among participating patients.

Risk factors	Adjusted OR	95% CI of OR		P value
		Lower limit	Upper limit	
**Age**				
**< 50**	Reference	-	-	
**≥ 50**	3.36	1.94	5.82	< 0.001*
**Place of living**				
**Cairo**	Reference	-	-	
**Other**	2.06	1.23	3.44	< 0.001*
**Indication for current admission**				
**General surgery**	Reference	-	-	
**Special surgery**	0.89	0.40	1.97	0.774
**General IM**	1.06	0.39	2.92	0.907
**Special IM**	1.44	0.71	2.94	0.314
**Liver/ GIT complaint**	4.21	1.78	9.94	0.001*
**History of PAT**				
**No**	Reference	-	-	
**Yes**	2.67	1.22	5.85	0.014*
**History of Blood transfusion**				
**No**	Reference	-	-	
**Yes**	1.66	0.90	3.03	0.104
**History of ASU admission**				
**No**	Reference	-	-	
**Yes**	1.41	0.83	2.41	0.206

Dependent variable: anti-HCV antibody status. Likelihood Ratio Chi-Square = 102.02, on 9 df; p-value < 0.001.

*Indicates statistical significance (p<0.05).

IM: Internal medicine, PAT: Parenteral anti-schistosomiasis treatment.

## Discussion

In this study, we described the HCV status and risk factors associated with anti-HCV antibodies among 500 patients admitted to the internal medicine and surgery Ain Shams hospitals in Cairo, Egypt, as well as among 50 HCWs from these two hospitals. We found a 19.80% prevalence of HCV antibodies among participating patients, which is high compared to available estimates among hospital patients from other countries that are typically below 5% [16–21], but in accordance with the previously observed high prevalence of HCV among Egyptian hospital patients [14,22]. In participating HCWs, we found a lower prevalence of 8.0%, although this is again higher than estimates from other countries [23–28]. In both groups, older age was strongly associated with positive HCV status. Among patients, we also found HCV positive status to be associated with living outside Cairo, being admitted for liver problems, having received PAT or a blood transfusion, and a previous admission to Ain Shams University hospitals.

The prevalence we found among HCWs (8.00%) is consistent with that previously estimated in Ain Shams university hospitals by Munier et al. (7.2%, 95% CI: 5.3%-10%) [14] and by Okasha et al. (8.0%, 95% CI: 6.7%-9.2%) [29]. The higher prevalence found among patients than among HCWs was also expected, due to the high prevalence of older individuals and frequent at-risk invasive medical exposure in this population, especially in the internal medicine hospital. However, we found a lower patient prevalence upon admission within the internal medicine hospital (28.8% (65/226), 95% CI: 22.9%-34.7%) than the prevalence estimated in 2008–2010 in the Munier study among patients of this same hospital (55.6% (75/135), 95% CI: 47.2%-63.9%) [14]. This may simply reflect the overall decreasing prevalence of HCV in Egypt, due in part to the aging of the cohort that was infected in the 1960’s, as well as to the implementation of infection control measures through the recently established National Committee for the Control of Viral Hepatitis.

The risk factors we found for patients are also in line with the findings of earlier epidemiological studies. Older age and history of PAT are well-known risk factors of HCV in Egypt [4]. HCV prevalence is known to vary according to the place of residence in Egypt, with a higher prevalence in rural governorates [30] where access to treatment and referral centres may also be more of an issue, explaining why we found living outside Cairo to be a risk factor. In addition, history of blood transfusion has been identified in 24.3% and needle reuse in 20.6% of HCV-positive cases in earlier studies [31,32]. It should be noted here that, to limit recall bias in our cross-sectional study, we did not collect information on the date when these procedures were performed, and therefore were unable to determine whether older exposures may have been more at-risk than more recent exposures, potentially reflecting progress in infection control.

The cross-sectional nature of our study also limits the causal interpretation of our results, as this design allows for reverse causation. In particular, the other procedures we found associated with HCV seropositivity in the bivariate analyses (endoscopy and gastric lavage) may not necessarily be predisposing factors for HCV infection. Rather, patients with chronic HCV liver disease could have specifically undergone these procedures for treatment of a complication such as hematemesis due to oesophageal varices, which is a common presentation for chronic HCV patients admitted to hospitals. The same inverse-temporal relationship goes for the association with admission for liver problems, which is probably a consequence of HCV. Finally, the association we found with history of ASU admission may be due to the fact that ASU hospitals are a referral centre for treatment of HCV liver disease. It could also reflect the fact that the population that ASU hospitals serve is different from populations from other public or private hospitals regarding their HCV prevalence. In the current study, out of 44 ever hospitalized patients with previous HCV diagnosis, 33 (68.75%) had a history of admission to ASU hospitals (p < 0.001).

In their occupational environment, HCWs are frequently exposed to multiple blood-borne pathogens, including HCV. After residents, nurses and physicians have been shown to be at highest risk of acquiring HCV infection during their daily practice [33]. Previously identified risk factors for HCV infection among HCWs include being female, certain types of profession, the type of ward they work in, the frequency and nature of invasive procedures they perform, the severity of injuries (if any) of the patients they tend and the viral load in HCV-infected patients in that facility [34,35]. Based on the literature, the major route of HCV acquisition in HCWs appears to be needlestick injuries [36]. In Egypt, the number of needlestick injuries was estimated at 4.9 per HCW per year [37], and has been shown to remain high even in recent years [38].

Due to the design of this study, only 50 HCWs were included, leading to a clear lack of statistical power that hindered our full understanding of their HCV status. In addition, in order to limit the length of the study inclusion questionnaires, we chose to focus on a few aspects of infection control practices and knowledge rather than use a longer though validated scale such as the Compliance with Standard Precautions Scale [39]. However, the risk factors we did find to be associated with HCV seropositivity in HCWs are in line with those reported in earlier studies.

Other limitations of our study include its potential lack of generalizability to other hospital specialties beyond internal medicine and surgery (e.g. intensive care unit or obstetrics), non-university hospitals and Egyptian hospitals located outside Cairo. Finally, the questionnaire data we collected may also have beeen affected by recall bias, possibly causing misclassification of past medical exposures among study participants.

## Conclusions

Over the last decades, many efforts have been implemented in Egypt to control the spread of HCV. In 2008, the National Committee for the Control of Viral Hepatitis developed a National Control Strategy for Viral Hepatitis and established HCV prevention and treatment programs [40]. The committee recommended prevention and education campaigns targeting the general population as well as healthcare workers (HCWs) [41]. Following successful negotiations for 99% discounted direct-acting antiviral agents prices [42], Egypt launched an ambitious national HCV treatment program aiming to treat over 250,000 chronically infected individuals per year, with the goal of achieving a national chronic infection prevalence of <2% by 2025 [43]. Finally, in October 2018, Egypt’s Ministry of Health launched the “100 Million Healthy Lives” initiative, which aimed at screening more than 52 million citizens for hepatitis C (HCV). The initiative ended in April 2019 after targeting more than 45 million citizens.

However, in order to fully control HCV in the Egyptian context, better understanding the role played by hospitals and the risks incurred for both hospital patients and HCWs remains crucial. This work may help in this regard, for instance by allowing to identify at-risk patient profiles upon hospital admission, for whom reinforced control measures should be implemented during their entire hospital stay. The importance of defining such criteria is underlined by the high rates of occult infections we found. This work also constitutes the first step in a larger follow-up study, during which both participating patients and HCWs were followed longitudinally in order to assess their risk of HCV acquisition due to exposure to at-risk medical procedures within the hospital.
